# FOXM1 predicts overall and disease specific survival in muscle-invasive urothelial carcinoma and presents a differential expression between bladder cancer subtypes

**DOI:** 10.18632/oncotarget.17394

**Published:** 2017-04-24

**Authors:** Sebastien Rinaldetti, Ralph Markus Wirtz, Thomas Stefan Worst, Markus Eckstein, Cleo Aaron Weiss, Johannes Breyer, Wolfgang Otto, Christian Bolenz, Arndt Hartmann, Philipp Erben

**Affiliations:** ^1^ Department of Hematology and Oncology, University Medical Center Mannheim, Medical Faculty Mannheim, University of Heidelberg, 68167 Mannheim, Germany; ^2^ Stratifyer Molecular Pathology GmbH, 50935 Köln, Germany; ^3^ Department of Urology, University Medical Center Mannheim, Medical Faculty Mannheim, University of Heidelberg, 68167 Mannheim, Germany; ^4^ Institute of Pathology, University Erlangen-Nuremberg, 91054 Erlangen, Germany; ^5^ Institute of Pathology, University Medical Center Mannheim, Medical Faculty Mannheim, University of Heidelberg, 68167 Mannheim, Germany; ^6^ Department of Urology, University of Regensburg, 93053 Regensburg, Germany; ^7^ Department of Urology, University of Ulm, 89075 Ulm, Germany

**Keywords:** FOXM1, muscle invasive bladder cancer, KI67, subclassification, survival prediction

## Abstract

Forkhead box M1 (FOXM1) is a late cell cycle gene that plays a crucial role in carcinogenesis and chemotherapeutic drug resistance. In this study, the impact of FOXM1 expression on patient outcome was investigated for the first time in formalin fixed and paraffin embedded (FFPE) samples of chemotherapy naïve muscle-invasive bladder cancer (MIBC) patients. Expression analyses were performed on the Mannheim cohort (n=84) and validated on the independent Chungbuk cohort (n=61). In a Cox’ proportional hazards model, a distinct FOXM1 expression cut-off dividing both cohorts in a ‘high-risk’ and ‘low-risk’ group has been determined. Multivariate analyses showed that FOXM1 is an independent risk factor for outcome prediction superior to the TNM system. The FOXM1 ‘high-risk’ group had a 4- to 7-fold increased risk of death (p<0.03) and presented further an overexpression of MKI67. Recent studies showed that MIBCs can be subclassified in breast cancer-like subtypes: basal, luminal and p53-like. Here we demonstrated that FOXM1 was differentially expressed between MIBC subtypes concordant to its subtype specific expression in breast cancer. Since the proto-oncogene FOXM1 is known to play an important role in cisplatin resistance and to be a promising drug target, this study supports FOXM1 as a crucial biomarker in the personalization of MIBC therapy and urges prospective translational studies.

## INTRODUCTION

Bladder cancer is one of the 10 most common malignancies worldwide with nearly 386,000 new cases and nearly 150,200 deaths per year [1]. While, muscle-invasive bladder carcinoma (MIBC) account only for about 30-40% of the cases, they present the poorest outcome [2]. Patients with metastatic carcinoma have a 5-year overall survival rate of approximately 20% [3]. Additionally, due to the high costs of transurethral resections, cystectomies, chemotherapies and the often necessary lifelong surveillance, bladder cancer is one of the most expensive cancer entities averaged over all US citizens [4].

The current standard of care in MIBC is radical cystectomy. High risk patients are frequently treated with a perioperative platin-based chemotherapy. At the moment, the diagnostic and therapy of MIBC suffers from at least two major problems. First, the therapy selection is heavily influenced by an insufficient clinicopathologic staging system for survival and therapy response prediction, as clinical understaging occurs in 46% of the cases [5]. Second, unlike in other tumor entities, there are no prognostic biomarkers established in the clinical routine. Yet, the search for biomarkers may improve prognostication and personalization of bladder cancer therapy and may pave the way to new targeted neoadjuvant therapy options [6]. In this context, several studies subclassified MIBC through gene expression profiling [7–9]. Those studies identified distinct molecular subtypes which correlated well with outcome and therapy response and were similar to the molecular phenotypes of breast cancer.

The forkhead box M1 gene is known to be involved in many of the hallmarks of carcinogenesis, e.g. cell cycle progression, angiogenesis, epithelial-mesenchymal transition, cell migration, genomic instability and formation of tumor metastasis [10–15]. Its expression is negatively regulated by the tumor suppressor gene TP53 and its downstream gene signature seems to be enriched in the SCC-like and Genomically Unstable bladder cancer subtype as defined by Sjödahl et al. [16–18]. FOXM1 is also associated with resistance development against numerous anti-cancer drugs (e.g. cisplatin, paclitaxel, docetaxel, gefitinib, epirubicin and trastuzumab) [19–25].

Meta-analyses showed that overexpression of FOXM1 was associated with poor prognosis in many solid cancers such as breast, gastric, non-small-cell lung, pancreatic and hepatocellular carcinoma [26–32]. FOXM1 has been shown to be overexpressed on mRNA level as well as on protein level in bladder cancer cells in comparison with normal urothelial cells [15].

This study investigates for the first time the impact of FOXM1 expression on outcome prediction and risk stratification in two independent MIBC cohorts. The Mannheim cohort includes patients treated exclusively by cystectomy in order to investigate the genuine cours of disease for patients with high versus low FOXM1 expression. The Chungbuk cohort includes patients with adjuvant cisplatin therapy in order to investigate the role of FOXM1 expression in the context of cisplatin resistance. Recent transcriptome expression studies showed the existence of molecular subtypes (basal, luminal and p53-like) similar to breast cancer molecular phenotypes.

In this context, FOXM1 was tested for differential expression in the luminal, basal and p53-like bladder cancer subtypes [7, 8]. In this study, the latter has been renamed as, non-luminal non-basal’ (NLNB) given the lack of validation concerning this MIBC subtype [33]. As FOXM1 is a druggable proto-oncogene, the elucidation of its impact on bladder cancer survival may contribute to a further personalization of future MIBC therapy [34].

## RESULTS

### Cohort characteristics

Clinicopathologic characteristics of the Mannheim and the Chungbuk cohort are listed in Table 1 and Table 2. The 10-year overall survival (OS), disease specific survival (DSS) and progression-free survival (PFS) of 84 patients with MIBC enrolled in the Mannheim cohort were 35%, 50% and 41% respectively. From the Chungbuk cohort (n=165) only patients with muscle-invasive carcinoma were selected, resulting in a cohort of 61 patients with a 10-year OS, DSS and PFS of 40%, 45% and 49% respectively. In the Mannheim cohort, patients were exclusively treated with radical cystectomy and lymphadenectomy in contrast to the Chungbuk cohort, where 43% of the patients received adjuvant systemic chemotherapy after radical cystectomy or transurethral resection (TUR). The risk stratification according to a high or low expression of FOXM1 resulted in an equal distribution of patient characteristics in both cohorts between risk groups, except for low grade tumors in the Chungbuk cohort, which correlated significantly with low FOXM1 expression (p=0.023). Comparing tumor stage and grade, the Mannheim cohort included more advanced disease features.

**Table 1:** Clinicopathologic characteristics of the Mannheim cohort (n=84): High risk versus low risk MIBCs according to FOXM1 expression

	Total	%	High Risk	%	Low Risk	%	p-values
**Cohort Characteristics**							
Cohort size	84		54	(64)	30	(36)	
median age	66		66		64		0.685
female	20	(24)	14	(26)	6	(20)	0.603
male	64	(76)	40	(74)	24	(80)	
Progress (n=76)	43	(57)	29	(62)	14	(33)	0.182
**TNM Staging**							
pT1	1	(1)	0	(0)	1	(3)	0.34
pT2	21	(25)	16	(30)	5	(17)	
pT3	47	(56)	29	(54)	18	(60)	
pT4	15	(18)	9	(17)	6	(20)	
pN+ (n=82)	31	(38)	18	(35)	13	(43)	0.483
cM+ (n=70)	7	(10)	6	(14)	1	(4)	0.236
**Grading**							
G2	17	(20)	8	(15)	9	(30)	0.086
G3	67	(80)	46	(85)	21	(70)	
**Additional Therapy (n=81)**							
NAC	1	(1)	1	(2)	0	(0)	1.0
AC	11	(14)	7	(14)	4	(14)	0.607

**Table 2:** Clinicopathologic characteristics of the Chungbuk cohort (n=61): High risk versus low risk MIBCs according to FOXM1 expression

	Total	%	High Risk	%	Low Risk	%	p-values
**Cohort Characteristics**							
Cohort size	61		10	(16)	51	(84)	
Median age	66		73		66		0.051
Female	13	(21)	4	(40)	9	(18)	0.198
Male	48	(79)	6	(60)	42	(82)	
Progress	20	(33)	3	(30)	17	(33)	1.000
**TNM Staging**							
pT2	31	(51)	4	(40)	27	(53)	0.740
pT3	19	(31)	4	(40)	15	(29)	
pT4	11	(18)	2	(20)	9	(18)	
pN+	14	(23)	4	(40)	10	(20)	0.222
cM+	6	(10)	0	(0)	6	(12)	0.577
**Grading**							
Low grade	19	(31)	0	(0)	19	(37)	0.023
High grade	42	(69)	10	(100)	32	(63)	
**Additional Therapy**							
Systemic chemotherapy	26	(43)	3	(30)	23	(45)	0.494

### FOXM1 is an independent risk factor for survival prediction

**Figure 1:**
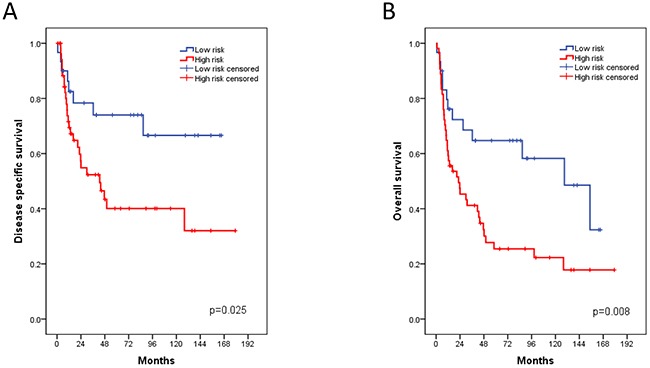
Kaplan-Meier plots of the Mannheim cohort for disease specific (A) and overall survival (B) associated with the FOXM1 risk stratification

The qRT-PCR analyses of the Mannheim cohort yielded a FOXM1 expression range from 30.7 to 35.5 (40-dCT) (median: 33.5) after normalization to the housekeeping gene CALM2. The FOXM1 cut-off value of 33.1 (40-dCt) showed best discrimination between a high risk and a low risk group. In order to determine the impact of FOXM1 expression on OS, DSS and PFS, we performed Cox's regression analysis, adjusted for grade, gender, TNM, therapy and low versus high FOXM1 expression (Table 3). The risk stratification between low and high FOXM1 expression allowed a concise discrimination between a high risk and low risk group. Multivariate analysis showed that patients with high FOXM1 expression have a 4- to 7-fold higher risk of death, by means of OS and DSS respectively (p<0.003). This risk stratification displayed higher HR than lymph node metastasis and tumor stage in the context of OS and DSS. Thus, FOXM1 presented an independent risk factor for MIBC survival prediction. The Kaplan-Meier estimates showed a 10-year DSS of high-risk versus low-risk patients according to FOXM1 expression of 40% versus 67% (p=0.017) and a 10-year OS of 22% versus 58% (p=0.04, Figure 1). However, this FOXM1 risk stratification gave no additional information concerning PFS (log rank test: p=0.09).

**Table 3:** Multivariate Cox regression analysis of DSS and OS for the Mannheim cohort after adjustment for standard clinicopathologic characteristics

	DSS			OS		
Cox regression analysis	HR	95% CI	p	HR	95% CI	p
FOXM1 high vs. low	6.76	2.36-19,35	<0.003	4.18	1.96-8.88	<0.003
pN+	5.89	2.38-14,57	<0.003	3.57	1.83-6.96	<0.003
pT1-2 vs. pT3-4	3.00	0.98-9,12	0.054		n.s.	

### Validation of the FOXM1 risk stratification in the chungbuk cohort

**Figure 2:**
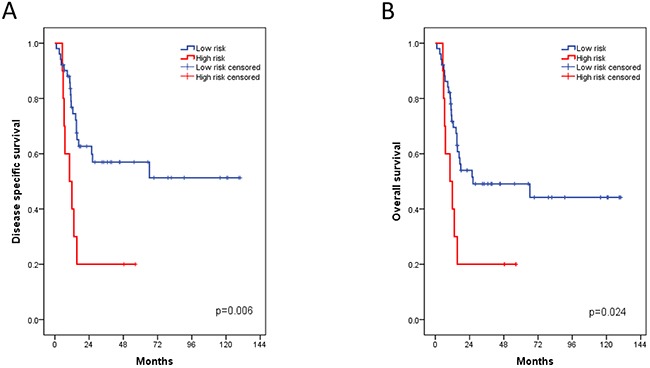
Kaplan-Meier plots of the Chungbuk cohort for disease specific (A) and overall survival (B) associated with the FOXM1 risk stratification

Despite of the differences in MIBC therapy and expression quantification methods, we managed as a first validation to define a clear FOXM1 cut-off in the Chungbuk cohort. Moreover the hazard ratios of the Chungbuk cohort for OS and DSS correlated with the risk stratification of the Mannheim cohort (Table 4). Indeed the FOXM1 log2 cut-off value of 9.7 (range: 7.1-11.6, median: 9.3) allowed a dichotomization of the cohort in a low risk group with low FOXM1 expression and a high risk group with high FOXM1 expression. In the multivariate analysis, adjusted for the same parameters as in the Mannheim cohort, the risk of death is up to 3 times higher in the high risk group for OS and DSS (p<0.03, Table 4). The Kaplan-Meier estimates showed a 4-year DSS of high-risk versus low-risk patients of 20% versus 57% (p=0.006) and a 4-year OS of 20% versus 49% (p=0.024, Figure 2). PFS showed no statistical significance in the log rank test (p=0.4). In both cohorts tumor grade (G2 versus G3) was not an independent risk factor whereas lymph node metastasis and tumor stage were strong survival predictors. FOXM1 was the only parameter that was found as an independent risk factor in the Mannheim and Chungbuk cohort for both OS and DSS (p<0.03).

**Table 4:** Multivariate Cox regression analysis of DSS and OS for the Chungbuk cohort after adjustment for standard clinicopathologic characteristics

	DSS			OS		
Cox regression analysis	HR	95% CI	p	HR	95% CI	p
FOXM1 high vs. low	3.27	1.36-7.87	0.008	2.87	1.22-6.75	0.016
pN+	3.29	1.45-7.48	0.004		n.s.	
pT2 vs. pT3-4	2.53	1.08-5.95	0.033	2.44	1.16-5.15	0.019
cM+		n.s.		4.65	1.81-11.94	<0.003

### Subtype specific expression of FOXM1

**Figure 3:**
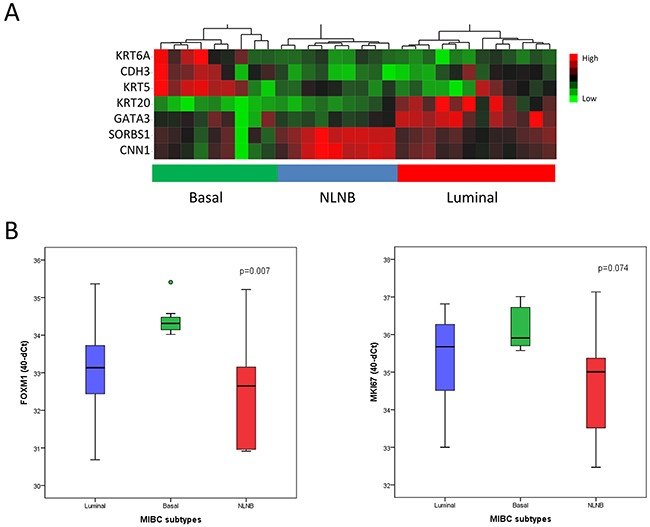
MIBC subclassification of the Mannheim cohort by Nanostring nCounter analysis (n=30) **(A)** Heatmap generated by unsupervised hierarchical clustering using a 7-gene signature. Luminal: KRT20, GATA3 (blue); basal: KRT6A, CDH3, KRT5 (green); non-luminal non-basal (NLNB): SORBS1, CNN1 (red). **(B)** Subtype specific expression of FOXM1 and MKI67 analyzed by qRT-PCR. Data are represented as median ±SD.

**Figure 4:**
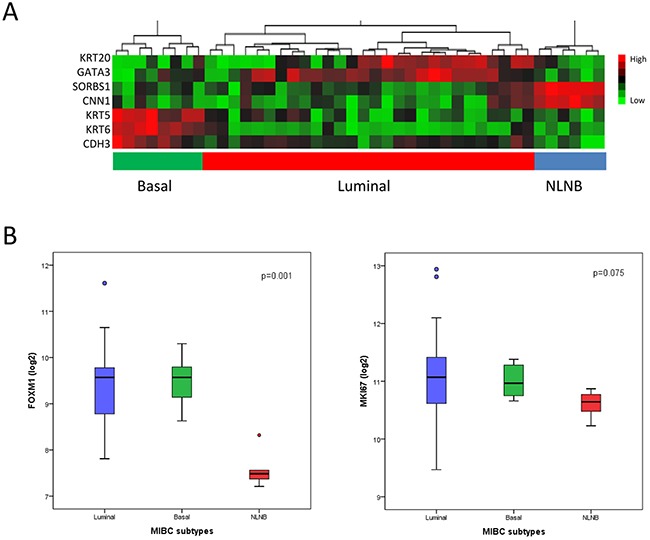
MIBC subclassification of the Chungbuk cohort by Illumina microarray analysis (n=42) **(A)** Heatmap generated by unsupervised hierarchical clustering using a 7-gene signature. Luminal: KRT20, GATA3 (blue); basal: KRT6A, CDH3, KRT5 (green); NLNB: SORBS1, CNN1 (red). **(B)** Subtype specific expression of FOXM1 and MKI67 analyzed by qRT-PCR. Data are represented as median ±SD.

Recent studies showed that muscle-invasive bladder cancer can be subclassified similarly to the molecular phenotypes of breast cancer into basal, luminal and NLNB subtypes. These subtypes have presented an impact on patient outcome and a differential expression of drug targets (e.g. ERBB2-3, FGFR1-4, ESR1) [7–9]. For MIBC subclassification, subtype specific genes from consensus data tested *in silico* for subtype enrichment were collected [33, 35, 36]. For both cohorts, we used a 7-gene panel for MIUC subtyping consisting in a curated luminal (KRT20, GATA3), basal (KRT5, KRT6A, CDH3) and NLNB (SORBS1, CNN1) gene signature. These genes formed consistent clusters throughout cohorts, as shown in Figure 3 and 4.

In the Mannheim cohort, FOXM1 showed a significant differential expression between subtypes, in particular a low expression in the p53-like subtype in comparison to the luminal and basal subtype (p=0.007, Figure 3B). These data were confirmed by the Chungbuk cohort, revealing as well a significant downregulation in the NLNB subtype (p=0.001, Figure 4B). The NLNB subtype is known to show good prognosis and to present immune infiltrative characteristics which may contribute to a dilution of the measured FOXM1 transcript level and thus distort the FOXM1 risk stratification [7, 37]. As a consequence the degree of immune infiltration was tested for the high and low risk group but showed no differential expression (Supplementary Figure 1, p>0.05).

### Differential expression of MKI67 between FOXM1 risk groups and MIBC subtypes

**Figure 5:**
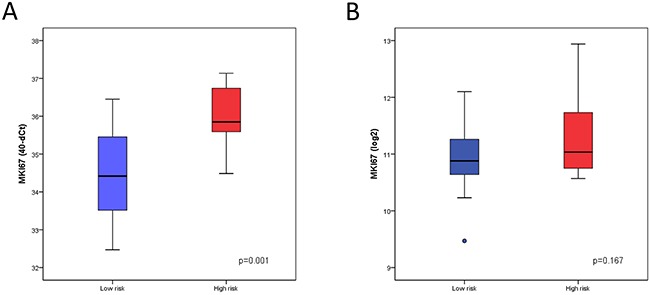
Differential expression of MKI67 between risk groups **(A)** Expression analysis of MKI67 determined by qRT-PCR in the low risk group (n=30) and high risk group (n=54) of the Mannheim cohort. **(B)** Expression analysis of MKI67 determined by Illumina microarray analysis in the low risk group (n=10) and high risk group (n=51) of the Chungbuk cohort.

As reported in previous studies FOXM1 regulates cell proliferation and invasion. Therefore we further investigated the differential expression of the proliferation marker MKI67 between the risk groups and MIBC subtypes (Figure 3–5). The expression of MKI67, determined by qRT-PCR, positively correlated with high FOXM1 expression in the Mannheim cohort (p=0.001). The same tendency could be observed in the Chungbuk cohort but without significance (p=0.167, Figure 5). The NLNB subtype, known to have a favorable outcome, presented the lowest MKI67 expression (Figure 4B). The Mannheim cohort confirmed this trend but without statistical significance (p=0.074, Figure 3B).

## DISCUSSION

The impact of the FOXM1 gene expression on survival has been investigated on a large scale of solid tumors and was associated with poor prognosis throughout these studies [26]. In the present study the prognostic value of FOXM1 has been evaluated for the first time in urothelial bladder cancer.

We stratified patients from two different cohorts according to their FOXM1 expression into two risk groups. Patients with a high expression of FOXM1 showed a 4- to 7-fold higher risk of death. The FOXM1 risk stratification even appeared superior to the TNM staging system for the prediction of DSS and OS in the Mannheim cohort. However, FOXM1 expression had no impact on predicting PFS in both cohorts. Nevertheless, FOXM1 allowed a consistent risk stratification and was confirmed to be an independent risk factor in multivariate Cox’ regression analysis. As mentioned above, high expression of FOXM1 had already been confirmed to be related to bad prognosis in many other cancer entities. From a biological point of view this seems plausible given the many roles of this protooncogene in cancerogenesis [26, 38].

In the Mannheim cohort 64% of the patients were ranked in the high risk group in contrast to the 16% of the Chungbuk cohort. Whether this is due to different quantification methods or therapy options between both cohorts remained unclear. However, this may partly be explained by the positive correlation of FOXM1 with cell proliferation, which enhances chemosensitivity [39]. Yet, exceeding a certain transcript level of FOXM1 may constitute a point of no return for resistance development. The Chungbuk cohort showed further 31% low grade tumors despite the selection of muscle invasive (>T2) tumors. As MIBC are rarely low grade, we cannot explain this high percentage.

In both cohorts tumor grade was not an independent risk factor. Tumor grade is known to have lower impact in muscle-invasive bladder cancer, as these tumors are mainly high-grade. Furthermore, radical cystectomy diminishes its predictive power [40–42]. In concordance with previous studies, lymph node metastasis and tumor stage were strong predictors in both cohorts [30]. Large scale studies showed MKI67 to be a valuable biomarker for outcome prediction in localized urothelial carcinoma but provided in advanced bladder cancer no additional information in comparison to tumor stage in advanced bladder cancer [43, 44]. Also in this study, MKI67 has not been retained in multivariate analyses.

In order to further investigate the translational impact of FOXM1 in MIBC, we analyzed its expression in the recent context of MIBC subclassification. We showed that FOXM1 is consistently enriched in the basal and luminal subtypes and suppressed in the NLNB subtype. These findings can be paralleled with data concerning the basal breast cancer subtype (triple negative), which also showed a FOXM1 overexpression and a poorer outcome [45–47]. Interestingly, our findings showed a considerable down regulation of FOXM1 in a MIBC subclass characterized by an activated signature of TP53 downstream genes [7]. Those have already been shown to be influential transcription factors in the downregulation of FOXM1 [16]. Thus, an overexpression of this proto-oncogene may be a surrogate marker for TP53 pathway inactivation, which is often associated with alterations common in aggressive urothelial carcinoma and correlates with the poor outcome of basal and luminal MIBC as described in recent studies [7, 16, 48, 49]. However, Choi et al. showed that the p53-like subtype had the highest rate of cisplatin non-responders. Considering the proliferation marker KI67, the NLNB subtype seemed to present a more quiescent subtype, which might have led to the described cisplatin resistance and thus present a FOXM1 independent resistance mechanism. On the other hand, this hypothesis needs further validation as the FOXM1 expression in the NLNB subtype may be diluted by inflammatory cells, though immune markers showed no enrichment in the FOXM1 low expression group in this study [37].

The *in vitro* knockdown of FOXM1 in a bladder cancer cell line showed a decrease of cell migration and proliferation [15, 45]. The same has been shown for the triple negative basal breast cancer cells [45]. In accordance with this, MKI67 has been positively correlated with FOXM1 expression in the Mannheim cohort (p=0.01), indicating a higher proliferation in MIBCs of the high risk group (Figure 5). Also of note is the presence of different FOXM1 isoforms, with FOXM1b exclusively expressed in cancer cells and FOXM1c with enhanced transforming potential [50]. As in this study all isoforms were covered by the different quantification methods, further investigations are warranted.

MIBC patients of our high risk group may profit from various direct FOXM1 inhibitors like siomycin A, thiostrepton and bortezomib [51, 52]. As luminal and basal bladder cancer subtypes are suspected to present a subtype specific overexpression of other well known drug targets like the FGFR, EGFR and ERBB gene families, the number of promising personalized therapy options rises for FOXM1 enriched MIBC [36]. The potential role in chemotherapy resistance against cisplatin, argues for further translational investigations on potential interactions of FOXM1 with current treatment options. As this biomarker improved survival prediction, FOXM1 may be integrated in future biomarker panels for molecular characterization of bladder cancer. Given the different quantification platforms between cohorts in this study, further prospective validation is needed. It has further been shown that elevated FOXM1 transcript levels in bladder cancer correlated with its protein expression. Thus, immunohistochemistry may also be a valuable tool in the detection of patients a risk [15]. The molecular phenotype may provide a superior tool for survival prediction than the TNM staging, given the heterogeneity of bladder cancer biology and the need for therapy personalization [53–56]. Since FOXM1 itself is praised to be a promising drug target in many solid tumor entities [21, 34, 38], translational studies are needed in order to implement FOXM1 in the race for MIBC therapy personalization.

## MATERIALS AND METHODS

### Patient population and specimen collection

Formalin fixed paraffin embedded (FFPE) tumor tissue samples were obtained from cystectomy of 84 muscle-invasive urothelial carcinoma patients (pT2-4, N0/1), who were treated exclusively with radical cystectomy in conjunction with bilateral lymphadenectomy (only 14% received a platin based combination therapy) at the University Medical Center Mannheim between July 1998 and January 2006. All patients gave informed consent. The retrospective analysis was approved by the relevant institutional review board under number 2016-814R-MA. The samples were evaluated for pathological stage according to the 2002 TNM classification of the American Joint Committee on Cancer. Histopathological parameters of cases were assessed by a pathologist specialized in uropathology (AH).

In order to validate our results *in silico*, we studied array expression data of 61 MIBC patients of the Chungbuck cohort (GSE13507). Tissue and histopathologic staging were obtained by cystectomy in muscle-invasive bladder cancer. MIBC patients were treated with at least 4 cycles of cisplatin-based chemotherapy as described before [57]. Patients with squamous cell carcinoma were excluded as they may distort clustering and deflect from the genuine pathophysiology of muscle-invasive transitional cell carcinoma. Grading of this cohort was assessed according to WHO grading classification 2004.

### Expression analysis of FOXM1 and MKI67 in the Mannheim cohort

RNA was extracted from 81 FFPE samples of MIBC patients according to a fully automated, high-throughput extraction workflow which runs on an Xtract XL liquid-handling robot (STRATIFYER Molecular Pathology GmbH, Cologne, Germany). One-step qRT-PCR was applied for the relative quantification of FOXM1 and MKI67 mRNA by using TaqMan quantitative RT-PCR. Calmodulin 2 (CALM2) was used as reference gene [58–60]. Gene expression has been assessed in duplicates by qRT-PCR using the SuperScript III PLATINUM One-Step quantitative RT-PCR System (Invitrogen, Karlsruhe, Germany) on a Stratagene Mx3005p (Agilent Technologies, Böblingen, Germany). FOXM1 expression analysis was performed with the following primers covering all isoforms (forward 5’-GACCACCTGGAGCCCTTTG-3’, reverse 5’-GATGTTGGATAGGCTATTGTTGATAGTG-3’, Tamra probe 5’- AGAAACGGGAGACCTGTGCAGATG-3’). MKI67 expression analysis was performed with the following primers (forward 5’-CGAGACGCCTGGTTACTATCAA-3’, reverse 5’-GGATACGGATGTCACATTCAATACC-3’, Tamra probe 5’-ACGGTCCCCACTTTCCCCTGAGC-3’). Ct values were normalized by subtracting the Cq value of the endogenous reference gene CALM2 from the Ct value of the target genes (ΔCt) [60]. Expression results were then reported as 40-ΔCq values which correlate proportionally with the mRNA expression level of the target genes.

### Statistical analysis

Clinico-demographic characteristics were compared with Fishers exact test, the Mann-Whitney U-test and the Kruskal-Wallis test. A distinct FOXM1 cut-off for risk stratification by means of survival prediction was determined by comparing iteratively the HR for different cut-off levels with the Cox proportional hazards model. The cut-off with highest HR was considered as appropriate for discrimination between a high risk and a low risk group. Kaplan-Meier estimates together with the log-rank test were used for survival analysis. The level of significance was <0.05. The primary endpoints were disease specific survival (DSS) and overall survival (OS) defined as death for any reason. Also progression-free survival (PFS) was recorded as time interval between cystectomy with lymphadenectomy and local or metastatic progression. Statistical analyses were performed with SPSS software version 20 (IBM, Armonk, NY, USA).

### Subclassification of MIBC patients and data validation

For MIBC subclassification, subtype specific genes from consensus data tested insilico for subtype enrichment were collected [33, 35, 36]. For both cohorts, we used a 7-gene panel for MIUC subtyping consisting in a curated luminal (KRT20, GATA3), basal (KRT5, KRT6A, CDH3) and p53-like (SORBS1, CNN1) gene signature. Patients were assigned to the different subtypes by the Ward unsupervised hierarchical clustering method using the JMP software version 12 (SAS Institute, Cary, NC, USA). The subtype specific expression of FOXM1 and MKI67 was verified by the Kruskal-Wallis test.

The MIBC subclassification was performed by the nCounter technology (Nanostring, Seattle, WA, USA) on a subgroup of the Mannheim cohort [61]. An amount of 100ng total RNA was used as input after quality control with qPCR and Nanodrop 1000 (Thermo Scientific, Wilmington, DE, USA). Preprocessing was performed by the nSolver Software 2.5 (Nanostring). The nCounter assay was normalized using the geometric mean of 6 reference genes and 6 positive controls. Reference genes were selected in order to cover low expression (G6PD, TUBB) as well as high expression genes (B2M, CALM2, GAPDH and RPL37A) with a Spearman correlation of at least 0.40 and showed a mean probe normalization factor of 1.3. Negative background substraction was performed by 8 negative controls.

Validation of MIBC risk stratification and subclassification was performed in silico on the Chungbuk cohort (GSE13507) based on Illumina human-6 v2.0 beadchip data of 61 MIBC patients. Preprocessing of array data was realized by the Illumina BeadStudio software using quantile normalization and log2 transformation. Only high grade tumors were selected for MIBC subclassification given the heterogeneity of tumor grade in the Chungbuk cohort. Indeed, it has been shown that molecular subclasses vary strongly between tumor grade [62].

The other authors declare to have no conflicts of interest.

## SUPPLEMENTARY MATERIALS FIGURES


