# Supplementary material for: FOXM1 predicts overall and disease specific survival in muscle-invasive urothelial carcinoma and presents a differential expression between bladder cancer subtypes
Source: Oncotarget. 2017 Apr 24;8(29):47595–606. doi: 10.18632/oncotarget.17394 (PMC5564590; doi:10.18632/oncotarget.17394)
Supplement: Supplementary file 1 [file oncotarget-08-47595-s001.pdf]

FOXM1 predicts overall and disease specific survival in muscle-invasive urothelial carcinoma and presents a differential expression between bladder cancer subtypes

SUPPLEMENTARY MATERIALS

SUPPLEMENTARY FIGURE

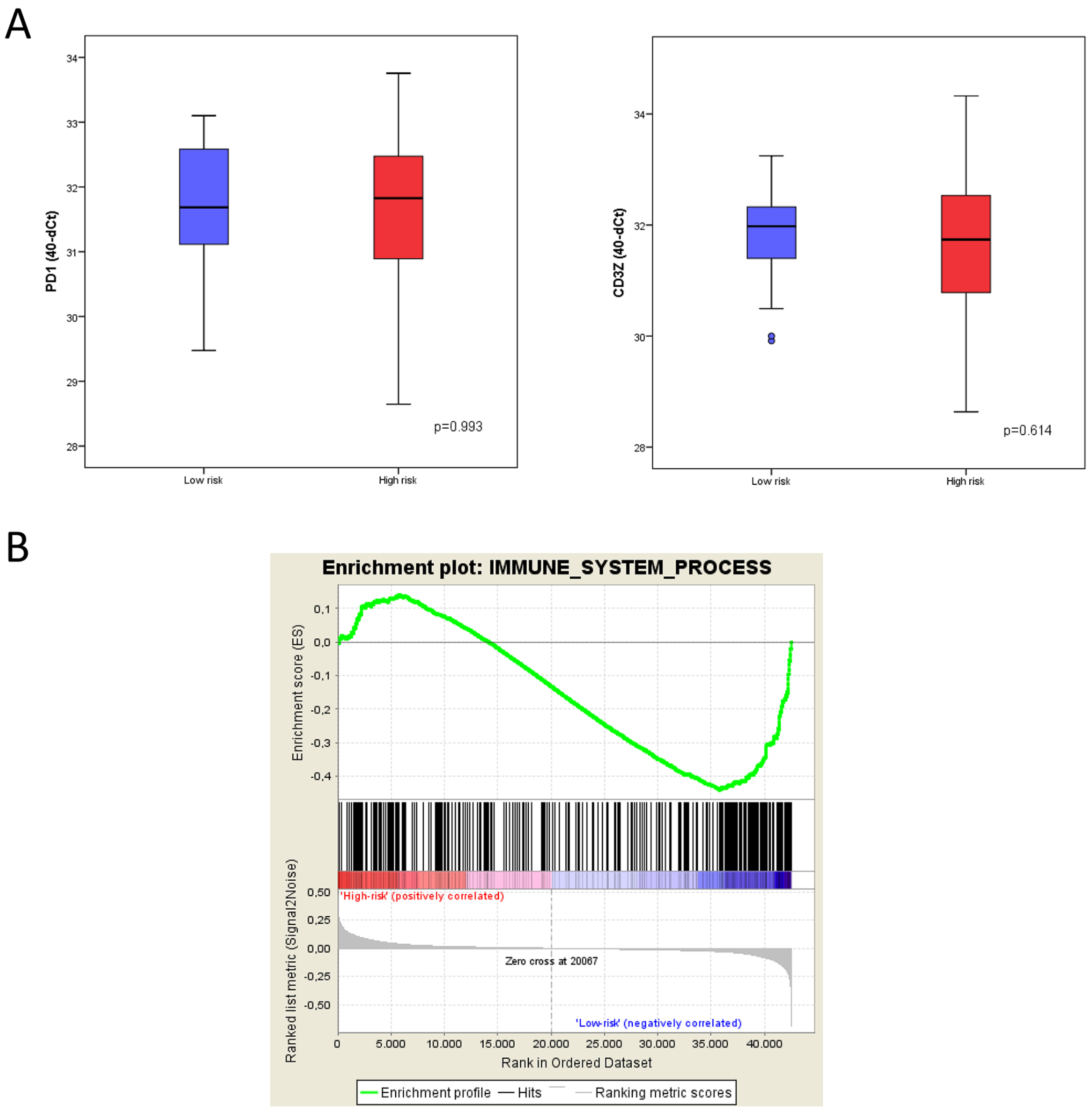

Supplementary Figure 1: Impact of immune infiltration on risk groups. (A) The immune infiltration markers PD1 and CD3Z showed no subtype specific expression between risk groups. **(B)** Gene set enrichment analysis by GSEA v2.2.3 (Broad Institute) of an immune signature from the Gene Ontology database showed no risk group specific enrichment (FDR=0.45, p=0.45).
